# Using Latent Selection Difference to Model Persistence in a Declining Population

**DOI:** 10.1371/journal.pone.0098126

**Published:** 2014-05-27

**Authors:** Mara E. Erickson, Christine Found-Jackson, Mark S. Boyce

**Affiliations:** 1 Department of Biological Sciences, University of Alberta, Edmonton, Alberta, Canada; 2 Alberta Environment and Sustainable Resource Development, Edmonton, Alberta, Canada; University of Kent, United Kingdom

## Abstract

Population persistence is a direct measure of the viability of a population. Monitoring the distribution of declining populations or subpopulations over time can yield estimates of persistence, which we show can be modeled as a latent selection difference (LSD) contrasting attributes of sites where populations have persisted versus those that have not. Predicted persistence can be modeled with predictor covariates to identify factors correlated with species persistence. We demonstrate how to model persistence based on changes in occupancy that can include adjustments for detection probability. Using a known historical distribution of the western grebe (*Aechmophorus occidentalis*), we adapted methods originally developed for occupancy modeling to evaluate how environmental covariates including emergent vegetation and human developments have affected western grebe persistence in Alberta. The relative probability of persistence was correlated with the extent of shoreline bulrush (*Scirpus lacustris*), which is important vegetation for nesting cover. We also documented that western grebe populations were less likely to persist on lakes in the boreal forest, primarily located on the northern boundary of the species' range. Factors influencing occupancy were different than those determining persistence by western grebes; persistence and occupancy were not correlated. Persistence was more likely on lakes with recreational development, reflecting reliance by grebes on the larger, fish-bearing waterbodies that also are attractive for lakeshore development. Unfortunately, the correlation with recreational development on Alberta's lakes puts grebes at risk for loss of brood-rearing habitats—primary threats to altricial birds—if steps are not taken to prevent disturbance to bulrush stands. Identifying factors related to the persistence of a species—especially one in decline—is a fundamental step in conservation management.

## Introduction

The distribution of threatened vertebrates can be affected by various threats that influence persistence of populations [1]. Likewise, identifying attributes of habitats where a species has persisted relative to habitats where it has disappeared should help to identify extinction threats for a species in decline. Documenting such spatial patterns of population persistence can be central to the development of effective conservation programs.

Persistence has been defined as the constancy of a species' presence at a site over time [2]. Unlike occupancy modeling which involves contrasting locations occupied by a species with those unoccupied at randomly selected locations [3], estimating persistence involves comparing a species' current distribution to its known former distribution. For a population declining in distribution and abundance, the current distribution is a sub-set of the species' original distribution of once-occupied sites, and locations can be classified as currently occupied versus those where populations have been extirpated.

MacArthur [4] developed analytical models of persistence contributing to the theoretical foundations of conservation biology, but only recently have statistical methods for analysis of persistence been considered. In a hypothetical system, Cross and Beissinger [5] used logistic regression to uncover mechanisms behind extinction or persistence among simulated PVA models for African wild dogs (*Lycaon pictus*). In this case, some model populations persisted and others did not, providing a binary classification appropriately analyzed using logistic regression. Empirical study of population persistence is seldom so simple. Species detection is usually recorded with little error, but failure to observe a species might be a consequence of true absence or simply a failure to detect the species at the time of a survey [6]. Therefore, presence/absence data frequently are burdened by an asymmetry of errors [7].

Occupancy analysis involves comparison of occupied and unoccupied resource units [3]. Resource selection functions typically use the logistic discriminant function to characterize selection by animals from a set of available resource units [8]. In contrast, in this application for persistence modeling we have two sets of selected resource units where selection has already occurred, but these selected resource units (lakes) differ in whether grebe populations have persisted or not. Here, the appropriate statistical framework is the latent selection difference (LSD) estimated using logistic regression to identify differences between lakes where the birds have been extirpated (0) versus those where they still persist (1) [9].

MacKenzie et al. [3] developed methods for incorporating detection probability into occupancy models, typically based on repeated surveys of the same sites. Analytically, our method for estimating persistence is similar to methods for estimating occupancy, although the baseline sample of originally occupied lakes for estimating persistence is likely to be very different from a sample of potentially occupied lakes used to estimate occupancy. MacKenzie et al. 's method for correcting for detection can apply to persistence modeling where we begin with an assemblage of populations (or subpopulations of a metapopulation) that can be monitored over time to document variation in persistence. By accounting for detection probability, more reliable estimates of population persistence can be obtained. By measuring attributes of populations that persisted versus those that were extirpated, we can identify correlates of persistence, or conversely, local extinction.

Land-cover change and human activity often equate to habitat degradation—the number one threat to persistence of bird species [10], [11]. Likewise, changes in land-cover, human activity, and climate change have been shown to influence persistence of populations of various plants [12], butterflies [13], fish [14], and mammals [15].

The western grebe (*Aechmophorus occidentalis*) is a migratory waterbird that occurs exclusively in North America [16]. This species no longer occurs at many of its historical breeding sites including several in British Columbia [17], California [18], and Alberta [19], [20], and has experienced declines on its wintering grounds [21]. The marked wintering-site declines might be in concert with a large distribution shift noted along coastal California [22]. Low power in detecting genetic differentiation between breeding colonies coupled with limited re-sightings of banded birds makes it difficult to ascertain true site fidelity for the western grebe [23]. However, consistent surveys over the past 40 years in Alberta and other jurisdictions with breeding populations suggest that the western grebe not only uses the same breeding lakes year after year, but the same nesting area within a lake as well, lending itself well to studies of persistence. For instance, reports from the late 1970s (J. Kristensen and W.R. Nordstrom, unpublished report) coupled with more recent surveys indicate that western grebes have been breeding at Cold Lake's Centre Bay at least 30 years [24]. The Wabamun Lake western grebe colony continued to use the same nesting area even after a Canadian National train derailment in 2005 resulted in over 700 000 L of oil spilling near the shore, most of which polluted the lake [25]. Knowledge of these sites and specific characteristics that can affect grebe persistence (e.g., type and density of vegetation, potential disturbance) in the face of expanding human use of the landscape will aid in identifying key features of western grebe habitats for focusing future conservation efforts. Known factors affecting occurrence of breeding grebes on a lake include protection from predation and anthropogenic disturbance, stable water levels, protection from wind, ample water depth around and within the colony, open-water access and prey availability, and an ice-free period to allow successful nesting [26]. Other research on the western grebe in Alberta has found that the species is more likely to occur on large lakes and less likely to occur on marsh-type (<3 m deep dominated by emergent macrophytes) lakes, and waterbodies surrounded by boreal forest vegetation [27].

From a sample of lakes where western grebes were known to occur in the past 40 years, we modeled population persistence using the latent selection difference (LSD) to identify differences in attributes between those lakes where the western grebe persisted and lakes where the species has disappeared. We hypothesize that factors known to be important to the western grebe, such as ample nesting habitat, prey availability, and adequate lake size, would be positively correlated with western grebe persistence, while factors that might cause disturbance to the birds during their breeding season (e.g. human activity and development) would be inversely correlated with persistence.

## Materials and Methods

### Western grebe surveys

**Figure 1:**
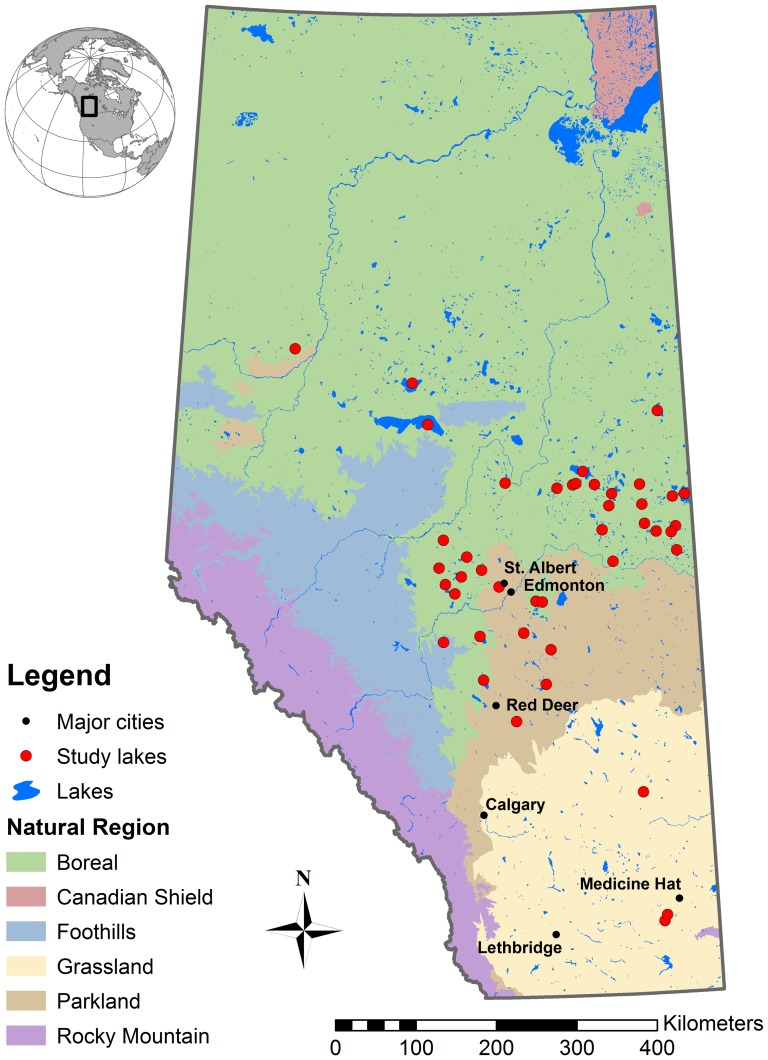
Locations of study lakes (*n* = 43) in Alberta, Canada where western grebes (*Aechmophorus occidentalis*) have been recorded since 1970.

We surveyed 43 publically accessible lakes for the presence of western grebes in the Boreal Forest (*n* = 34), Central Parkland (*n* = 6), and Grassland (*n* = 3) regions of Alberta (Fig 1) up to three times (roughly once every 30 days) from late May through late August of 2008 and 2009, and we documented human developments, shoreline/emergent/backshore vegetation, and other lake characteristics perceived to be important for the western grebe.

Since at least 1970, the western grebe has been documented on each of the 43 lakes that we monitored with 21 of these once supporting breeding populations of grebes. We attempted to census all lakes in Alberta where the western grebe has been documented historically and we included all known major breeding colonies since 1970. Historical surveys primarily were conducted by boat and occasionally by aerial surveys.

Our presence-absence surveys for western grebe primarily were conducted by boat or kayak. Lakes were stratified by size to adjust effort for surveying. Small lakes (<5 km^2^) could be surveyed by kayak, while medium (5–50 km^2^) or large (>50 km^2^) lakes required motor-powered watercraft. We surveyed along the shoreline (20–200 m out) depending on water depth and visibility, while scanning both between the shore and lake, as well as to the middle of the lake. Larger lakes required additional survey transects in the middle of the lake.

A small proportion of lakes were initially surveyed by air (fixed-wing or helicopter) due to size, or with spotting scopes if conditions were too poor for kayaks (e.g. strong winds), but were also surveyed by surface surveys later in the season. Spotting scopes only were used on small lakes where the entire lake could be viewed. A Pentax spotting scope (60×) was set on a tri-pod at various points around the lake, and the area was scanned using fixed points on the opposite shoreline as start/stop points at which to begin/end the next survey point. The entire lake was surveyed in this manner. To ensure consistency between this and historical western grebe surveys, all current surveys (both surface and aerial) followed techniques used by the Fish and Wildlife Division of Alberta Environment and Sustainable Resource Development, as outlined by the B.C. Resources Inventory Committee [28], and were conducted under the approval of the University of Alberta Animal Care and Use Committee. Lakes were considered “occupied” if western grebes were seen or heard during any of the surveys. Lakes were considered “breeding” lakes if either nests or young were observed during any of the surveys. Western grebes overlap the distribution of the similar Clark's grebe (*A. clarkii*) at 3 of our study lakes in the Grassland Natural Region of southern Alberta, but we did not detect any Clark's grebes during our study.

Using a standard detectability sampling scheme where *n* lakes were surveyed *K* times [3], lakes were surveyed up to three times during the season. Three surveys generally is recommended as the minimum number of repeat visits for a study design where detectability is estimated [3] and were considered sufficient due to the gregarious and conspicuous behaviour of the western grebe. (See Appendix S1 for 2008 survey dates and methods; complete western grebe observational data were submitted to and are available from the Alberta Fisheries & Wildlife Management Information System, FWMIS).

Four assumptions proposed by MacKenzie et al. [3] were necessary for this presence/absence/detectability study: 1) Survey units were closed to changes in presence on a lake, i.e., no immigration/emigration during the survey season. Western grebes initiate moult after nesting and tend not to fly once they are established on a lake during their breeding season [29], [30], [31]. Therefore, it was assumed that resource units (lakes) were closed to movement during the time of surveying (May-August). 2) The probability of persistence was constant across sites (or was modeled). 3) The probability of detection was constant across sites (or was modeled). We addressed assumptions 2 and 3 by explicitly modeling the influence of covariates for detection (i.e., date of survey and proportion of shoreline bulrush, *Scirpus lacustris*) and persistence (i.e., lake and vegetation characteristics). 4) Detection of grebes at a site was independent of that site's detection history. Observers often differed from survey to survey to reduce bias associated with observer skill, detection, and detection history.

### Model covariates

We recorded the extent (m) of bulrush along the shoreline because western grebes usually nest in bulrush [32]. Surveys were conducted from the water, 20–400 m from shoreline, with the distance to shoreline dependent upon water depth and visibility. Julian date of each survey was recorded and used as a detection covariate because western grebes might engage in more or less conspicuous behaviour depending on their breeding and moult status during the breeding season. Surveys were conducted between 0700 and 1600 hours, although time of day was not included as a detection covariate because the western grebes' behavior/use of the lake does not change substantially during the day-time period.

In the geographical information system (GIS) ArcMap [33], we used a combination of georeferenced aerial photography and satellite imagery (ranging from years 1999–2008; 0.5–1.0m resolution) as a reference to digitize and calculate the shoreline perimeter for 40 of 43 study lakes. An existing GIS layer of Alberta's lakes obtained from Alberta Environment and Sustainable Resource Development (AESRD) was used to digitize the shoreline perimeter of the remaining three lakes (Cold Lake, Lac la Biche, Winefred Lake). Alberta images were obtained from AESRD, and Saskatchewan images (for Cold Lake) were purchased from Information Services Corporation (ISC) Geomatics Distribution Center in Regina, Saskatchewan. In the case of a choice between resolution and age of photo, resolution took precedence, because the photos were used primarily as a reference for shoreline digitation, whereas ground-truthed data were used to digitize other variables such as shoreline development and emergent vegetation.

We digitized and quantified emergent bulrush along the shoreline using our ground-truthed data in combination with digital aerial photography. We then calculated the proportion of shoreline with bulrush stands. A 500-m buffer surrounding the lake was analyzed for human development and vegetative features. This buffer was used to characterize the dominant land-use surrounding the lake as well as to remain consistent with methods for assessing waterbird/fowl occupancy in Found et al. [27]. Anthropogenic development was digitized within this buffered zone using ground-truthed data and digital aerial photography, while terrestrial vegetation was quantified from an existing GIS land-cover classification raster layer [34]. Because 80–100% of western grebe diets are comprised of fish, we incorporated a measure of prey availability. The number of fish species in each lake was obtained from the Alberta Fish and Wildlife Management Information System and used as a proxy for prey availability because fish abundance data were not available for all study lakes.

Covariates were log-transformed if necessary to obtain approximate normality of distribution [35], [36], and examined for multicollinearity. If covariates were highly correlated (|*r*|>0.65), we retained the covariate with the highest predictive ability according to the analysis of single variables [35] (Table 1).

**Table 1:** Model covariates collected/generated from the 2008 and 2009 field season to identify correlates of western grebe persistence and detection probability of the western grebe in Alberta.

Covariate Name	Description	Category
***Develop***	**Proportion of human development in 500m buffer surrounding lake (log_e_ transformed) (% of buffer)**	**Buffer_anthropogenic**
*Con*	Proportion of coniferous forest in 500 m buffer surrounding lake (% of buffer)	Buffer_vegetation
*Dec*	Proportion of deciduous forest in 500 m buffer surrounding lake (% of buffer)	Buffer_vegetation
***Forest***	**Proportion of total forest in 500 m buffer surrounding lake (% of buffer)**	**Buffer_vegetation**
*Agr*	Proportion of agriculture in 500 m buffer surrounding lake (% of buffer)	Buffer_anthropogenic
*SA*	Lake surface area (km^2^)	Lake
*MaxDepth*	Maximum lake depth (m)	Lake
*MeanDepth*	Mean lake depth (m)	Lake
***Shore***	**Length of lake perimeter (km)**	**Lake**
***Fish***	**Number of fish species in lake (absolute value)**	**Lake**
*ShoreDevelop*	Proportion of human development along shoreline (%)	Anthropogenic
*RecIndex*	Recreational index of lake (0 = low; 1 = hi)	Anthropogenic
*Eveg*	Proportion of total emergent vegetation along shoreline (%)	vegetation
*TotEveg*	Proportion of total emergent vegetation in lake (%)	vegetation
*TotRush*	Proportion of bulrush (*Scirpus lacustris*) in lake (%)	vegetation
***Rush***	**Proportion of bulrush along shoreline (% of shoreline length)**	**vegetation**
***Date***	**Julian date of survey**	**Date**

Covariates listed in **bold** were included in LSD persistence models after a univariate analysis and multicollinearity screening.

### Persistence models

Alternative biologically plausible models were assessed using AICc for small sample size. AICc is an information-theoretic approach that maximizes the variation explained by a model while penalizing for the number of parameters in that model [37]. Models with lower AICc values are considered to be better explanations of the data relative to other models in the set. In addition to alternative models that explore model covariates separately as well as in combination to determine the best fit for the data, we included both a global model (all covariates) and a null model (no covariates) in the model-selection process (Table 2).

**Table 2:** *A priori* models used to identify correlates of western grebe persistence using an information-theoretic approach.

Model	Category	Description (model covariates)
1	Lake	*Shore*
2	Lake	*Fish*
3	Lake	*Shore + Fish*
4	Vegetation	*Rush*
5	Buffer	*Develop*
6	Buffer	*Rush*
7	Buffer	*Develop + Forest*
8	Lake + Vegetation	*Shore + Fish + Rush*
9	Lake + Buffer	*Shore + Fish + Develop*
10	Lake + Buffer	*Shore + Fish + Forest*
11	Vegetation + Buffer	*Rush + Forest + Develop*
12	Global	*Shore + Fish + Rush + Forest + Develop*
13	Null	*(no covariates)*

See Table 1 for covariate definitions.

We first modeled the relative probability of persistence independent of detection using the exponential form of the latent selection difference [9], [38], [39]: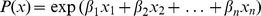 where *P*(***x***) is proportional to the probability of persistence on a lake, *β*_1_…*β_n_* are coefficients estimated from the data using logistic regression software, and ***x*** = *x*_1_, *x*_2_,…, *x_n_* are the values of the independent predictor covariates. The exponential form of equation 1 is the correct LSD model if we assume that predictor covariates are normally distributed [40]. An assumption for LSD is that the availability structure is the same (or similar) for both sets of lakes [9]; in fact, the underlying availability is essentially identical [27].

We compared the top four persistence models (those exhibiting similar support) while including the effect of detection error with the covariates *Rush* and *Date* (see Table 1 for covariate definitions). Emergent vegetation, especially bulrush, along the shoreline can provide cover for grebes, thus affecting the observer's ability to detect the birds. The date of survey also might affect detection probability due to the birds' varied use of the lake during the season (e.g., open-water foraging vs. use of bulrush beds), especially on breeding lakes. Model fit was assessed using a goodness-of-fit test in the program Presence 2.4 [3].

Finally, to illustrate the distinction between persistence and occupancy, we compared estimates of occupancy for each lake based on the western grebe occupancy model developed by Found et al. [27] with estimates of persistence from our top-performing persistence model. Occupancy was modeled by contrasting lakes where western grebes were present vs absent from a random sample of all lakes in the area, whereas our persistence model contrasted attributes of lakes that had been occupied since 1970 but were present or absent now. Specifically we assessed the correlation between the probability of occupancy and the relative probability of persistence of western grebes for each study lake. Statistical analyses were conducted using Stata 10.0 [41] and Presence 2.4 [42].

## Results

### Presence-absence surveys

At the end of the survey period, western grebes persisted on 27 (63%) of the original 43 lakes known to support grebes during the past 40 years. Evidence of breeding (presence of nests or young) was noted on ten of the original 21 breeding lakes, with established nesting colonies on eight of those ten.

### Persistence models

The global persistence model had the lowest AICc score and highest model weight (*w_i_* = 0.66) of all candidate models, followed by three models with ΔAICc<4 and a combination of the covariates *Rush*, *Forest*, and *Develop* (Table 3). Neither the number of fish species in a lake nor shoreline length were included in the top-performing persistence models.

**Table 3:** Comparison of top candidate LSD models, number of parameters, AICc values and differences, model likelihoods, model weights, -2 log likelihood scores for models identifying correlates of western grebe persistence in Alberta, Canada.

Model	*K*	ΔAICc	Model Likelihood	*w_i_*	-2LL
*Global*	6	0.00	1.00	0.66	34.77
*Develop*	2	3.40	0.18	0.12	41.11
*Rush + Forest + Develop*	4	3.62	0.16	0.11	46.08
*Develop + Forest*	3	4.78	0.09	0.06	44.93
*Shore + Fish + Develop*	4	6.42	0.04	0.03	44.14

After evaluating the top four models with the inclusion of detection covariates, there was a shift in the top-performing models as compared to the results without detection error (Table 4). Models containing the covariate *Shore* resulted in non-convergence and overdispersion; therefore, this covariate was excluded from the selection process. The global model ψ(*Rush + Forest + Develop*) p(*Rush + Date*) showed good fit to the data (test statistic = 1.027, c-hat = 0.97, *P* = 0.48), suggesting that the more parsimonious alternative models could adequately explain the data.

**Table 4:** Comparison of top candidate LSD models, number of parameters, AICc values and differences, model likelihoods, model weights, −2 log likelihood scores for models identifying correlates of western grebe persistence in Alberta, Canada incorporating detection error.

Model	*K*	ΔAICc	Model Likelihood	*w_i_*	-2LL
Ψ (*Develop*) p(.)	3	0.00	1.00	0.19	111.17
Ψ (*Develop*) p(*Rush*)	4	0.51	0.77	0.15	109.25
Ψ (*Rush + Forest* + *Develop*) p(.)	5	0.71	0.70	0.14	106.88
Ψ (*Rush + Forest + Develop*) p(*Rush*)	6	1.17	0.56	0.11	104.63
Ψ (*Develop + Forest*) p(.)	4	1.38	0.50	0.09	110.12

Models with a constant detection probability or those containing *Rush* as a detection covariate performed better than those with *Date* or *Date+Rush* (Table 4). Overall, the amount of development in a 500-m buffer surrounding the lake (β = 0.85; SE = 0.35) as well as the amount of bulrush along the shoreline (β = 3.56; SE = 2.09) were positively related to western grebe persistence, while the amount of forest in a 500-m buffer surrounding the lake (β = −2.41; SE = 1.98) was inversely related to western grebe persistence. As a detection covariate, *Rush* was inversely related to detection probability (β = −1.94; SE = 1.31). Based on 95% confidence intervals, the effects of bulrush and forested buffer were weak although the effects remained consistent across models in the set.

Even though our persistence model based on the LSD used statistical procedures similar to those used for estimating occupancy [3], the probability of occupancy for each lake used by western grebes calculated using the model of Found et al. [27] was not correlated with the relative probability of persistence of western grebes on the 43 lakes throughout Alberta (*r* = 0.0165, *P* = 0.9164).

## Discussion

Here we demonstrate a novel way to estimate persistence using LSD to contrast between occupied sampling units (lakes) versus those previously occupied. All lakes had supported grebes previously, but only a subset of those still support grebes now. Mechanistically this is similar to estimating occupancy where occupied units are contrasted with a sample of unoccupied resource units. The lack of correlation between persistence and occupancy of western grebes on lakes was not surprising, given that the underlying data (i.e. persistent/non-persistent vs. used/unused) were different. This illustrates the importance of study design in characterizing patterns of distribution and occupancy.

Ideally we would like to estimate persistence based on occupancy patterns documented year after year, an analysis that would lend itself to proportional hazards analysis such as the Andersen-Gill method [43]. Even though such data were not available, we still can document the outcome of differential persistence among sites based on the final distribution of birds after 40 years of exposure to extinction risks. Because western grebes tend to occupy the same lakes year after year, especially those designated as breeding lakes, this type of persistence modeling focusing on historically occupied sites can reveal factors contributing to the decline of the species in Alberta. Furthermore, for those lakes in our sample where grebes were lost, re-establishment of a breeding colony has not been documented.

### Detection error

Although western grebes are conspicuous birds, having the potential to adjust for detection adds considerable generality to the technique used in this study. The date of the survey was included as a potential source of detection error because the species might exhibit varying degrees of conspicuousness throughout the season, depending on breeding behavior. Similarly the amount of bulrush along the shoreline provides cover for grebes, and thus affects detection.

According to the persistence models, *Date* did not contribute appreciably to variation in detection probability, suggesting that the time of season when surveys were conducted for western grebes did not affect the observer's ability to detect the bird. *Rush* emerged as a possible source of detection error. However, the effect size of this covariate was weak, suggesting that current survey methods for western grebe were sufficient for detecting the species.

### Model covariates in relation to western grebe persistence

In all top models that included *Forest*, this covariate was inversely associated with western grebe persistence, suggesting that the grebes are disappearing more rapidly on lakes in the boreal forest on the edge of the species' range. Such shifts in distribution might be attributable to climate change [44], but we do not fully understand why local extinctions of western grebe are occurring on the edge of their range. Range contraction is not inevitable in a declining species [45], and this appears to be happening to the western grebe. The majority of the western grebe's range in North America occurs in drier grassland or sagebrush regions [16], whereas the extreme northern boundary of the species' range is in boreal forest. Breeding populations of white-winged scoter (*Melanitta fusca*) and lesser scaup (*Athya affinis*) also are declining in the boreal lakes of Alberta, and likewise we do not understand the reasons for these declines [46], [47], [48]. Still, the remaining boreal lakes in Alberta on which grebes have persisted continue to support important breeding colonies.

*Rush* was positively correlated with western grebe persistence. Bulrush is used for nest anchoring and construction [16], [32], [49], [50], and therefore serves as an important component of habitat. Ample emergent vegetation is needed to establish a nesting colony and serves as protection from wave action or other disturbances [51], [52]. Damage to or elimination of this vegetation along the shoreline (e.g., beach development) reduces the availability of colony locations and nesting material. For example, snowmobiling in reed beds and bulrush stands during winter shears off the old growth, rendering it useless for the birds when they return to breed the following spring [53]. Sensitivity to habitat disturbance/loss has been documented in other grebe species. For instance, the conversion of wetlands to pasture and agricultural land contributed to the decline and eventual extinction of the Colombian grebe (*Podiceps andinus*) [54]. In addition to loss of habitat due to recreational development (as well as natural causes), other anthropogenic influences, such as introduction of non-native fish species that both out-competed and preyed upon the Atitlán grebe (*Podilymbus gigas*), has been cited for that species' demise [55].

The covariate *Develop* was positively related to the persistence of western grebes. Similarly, Found et al. [27] found that the occurrence of western grebes in the northeastern boreal region of Alberta was associated with recreational activity. They attributed this relationship to possible habituation (also see [56]) as well as a tendency for humans to select lakes with the same characteristics that grebes select (i.e., deep water, presence of fish, medium to large lakes). For example, although western grebes persisted on lakes developed for recreation, these lakes also had a larger shoreline perimeter and a greater number of fish species. Therefore, it is difficult to separate grebe persistence on lakes with human development from human selection of preferred grebe habitats. Although there is still cause for concern because human developments sometimes result in the reduction of emergent vegetation [57], and therefore might affect persistence over time, the existence of lake cottages and other shoreline development might not have negative consequences so long as western grebe habitats remain intact and breeding sites are respected by boaters and other lake-users during the nesting period.

### Alternative explanations for western grebe persistence

Patterns of western grebe persistence, prey availability, and lake size lend support to alternative explanations of grebe persistence. Although information-theoretic methods of model selection can suggest what might be responsible for the amount of deviance from a model, it may not encompass all biologically plausible alternatives. For instance, the number of fish species was not included in the top models according to the AICc selection process even though it is known that the western grebe is almost exclusively piscivorous, and at some lakes the birds occupy top trophic positions [58]. However, all study lakes (but one) were currently fish bearing, suggesting that the species richness of fish might not affect persistence so long as an appropriate prey base exists. Examining the abundance of fish also might provide better information on the role of fish in the persistence of western grebe populations.

Persistence models containing shoreline length tended towards non-convergence and/or overdispersion in the model selection process. Therefore, this covariate was not included in the final models. However, all major breeding colonies occurred on lakes with a shoreline perimeter of at least 40.9 km; therefore, larger lakes provide conditions allowing persistence, at least by breeding grebes. These conditions might include more pockets of undeveloped shoreline; or, the greater lake surface area (which was correlated with shoreline perimeter and therefore not included in the analysis of western grebe persistence) might provide better, less disturbed, open-water foraging. Non-breeding grebes (i.e., no evidence of nests or young) were documented on even the smallest study lakes (e.g., Angling Lake, 9 km shoreline perimeter).

Other instances of low persistence might relate to direct disturbance. Common forms of nest-site disturbance during the breeding season include waves and wakes from boats and personal watercraft that can swamp nests. Boating and other recreational activity near the colony can provoke adults to make rushed exits leaving eggs vulnerable to exposure and accessible to predators such as ring-billed gulls (*Larus delawarensis*), black-billed magpies (*Pica pica*), and American crows (*Corvus brachyrhynchos*)—species common in areas with high anthropogenic disturbance. Adults might abandon unhatched eggs if disturbed during the hatching period [16]. Re-nesting is possible [59], but recruitment success is not guaranteed if chicks do not fledge early enough to develop fully before migration. Consequently, disturbance can reduce already low recruitment rates and might contribute to decreased persistence. Additional studies on the relationship between different types of anthropogenic activity and the details of disturbance to western grebes are warranted to address this issue.

Declines and distribution shifts on western grebe wintering grounds also might affect persistence on Alberta lakes. Because the wintering range is extensive (along the Pacific coast and in the Gulf of Mexico), changes in wintering distribution [22] could be reflected in subsequent breeding locations. However, because declines have been noted in several breeding locations outside of Alberta [17], [18], it is unlikely that the birds are simply migrating to new or different breeding sites, and thus we are seeing a true decline in persistence across the species' breeding range.

### Conservation actions

Because the western grebe has been recommended for threatened status in Alberta, and in light of its population declines elsewhere, we believe that it is important to pinpoint strategies to promote the persistence of this species. Of the patterns emerging from our analysis, maintaining shoreline vegetation is the one most amenable to management action. Other studies have recommended implementing a buffer zone around breeding colonies during the breeding season [19]. This would lessen the effects of wakes created by motor boats, and eliminate human disturbance from non-powered craft on both developed and non-developed lakes alike. Because the same lake (and, at breeding lakes, the same nesting site) often is used by grebes year after year, we suggest this buffer zone should remain during winter months to keep nest sites intact. Prohibiting activities that might scour or damage old-growth emergent vegetation (e.g., snowmobiling over the vegetation) will ensure vegetation is available the following spring for colony establishment.

Bulrushes and other natural emergent vegetation should remain intact, and not be removed for cottage or shoreline development. Not only will this benefit grebes, but also other colonial waterbirds that use emergent vegetation for nesting (e.g., red-necked grebes (*Podiceps grisegena*), Forster's tern (*Sterna forsteri*), Franklin's gull (*Larus pipixcan*)) and fish species that use aquatic and emergent vegetation for spawning and foraging such as northern pike (*Esox lucius*). Also, emergent vegetation is an indicator of a healthy aquatic environment, serving as habitat for species like those listed above while reducing shoreline erosion, and providing a more natural, esthetically pleasing shoreline [57].

Other detrimental effects to grebes and their habitats such as predation or nest damage from storms are not as easily controlled through management practices. Therefore, a concerted effort to encourage readily implemented, practical solutions are essential to maintain ample nesting habitat, minimize disturbance, and ensure persistence of the western grebe.

### Conclusions

Population persistence has been a fundamental target of conservation biology. Mace et al. [44] assert that features related to persistence (e.g., range contraction, abundance declines) are more likely to be influenced by anthropogenic pressures and habitat alteration than ecological or life history traits of a species. Sound methods for analyzing the factors contributing to the persistence of a species are necessary for effective conservation.

## Supporting Information

Appendix S1 **Dates and survey methods (Motored boat, kayak or canoe, spotting scope) for 2008 western grebe presence/absence surveys in Alberta.** ^1^Survey completed within week of specified date ^2^Colony check/nest count ^3^Survey conducted mid-July by Alberta Environment and Sustainable Resource Development
(DOCX)
