# Supplementary material for: Using Latent Selection Difference to Model Persistence in a Declining Population
Source: PLoS One. 2014 May 27;9(5):e98126. doi: 10.1371/journal.pone.0098126 (PMC4035306; doi:10.1371/journal.pone.0098126)
Supplement: Appendix S1 — Dates and survey methods (Motored boat, kayak or canoe, spotting scope) for 2008 western grebe presence/absence surveys in Alberta. 1Survey completed within week of specified date 2Colony check/nest count 3Survey conducted mid-July by Alberta Environment and Sustainable Resource Development (DOCX) [file pone.0098126.s001.docx]

| **Lake** | **Date 1** | **Method 1** | **Date 2** | **Method 2** | **Date 2** | **Method** |
| --- | --- | --- | --- | --- | --- | --- |
| Angling | 10-Jun | boat | 9-Jul | boat | 6-Aug | boat |
| Baptiste | 17-Jul | boat | 5-Aug | boat | 25-Aug | boat |
| Beaver | 16-Jun | boat | ----- | ----- | ----- | ----- |
| Big | 24-May | kayak | 20-Jun | kayak | 9-Aug | boat |
| Blood Indian Reservoir | 26-Jun^1^ | boat | ----- | ----- | ----- | ----- |
| Brock | 27-May | kayak | 20-Jul | kayak | 25-Aug | boat |
| Buck | 26-Jun^1^ | boat | 2-Aug | boat | 20-Aug | boat |
| Buffalo | 26-Jun^1^ | boat | 18-Jul | boat | 13-Aug | boat |
| Cardinal | 19-Jun | kayak^2^ | ----- | ----- | ----- | ----- |
| Coal | 3-Jun | kayak | 3-Jul | boat | 13-Aug | boat |
| Cold | 2-Jul | helicopter | 29-Jul | boat | 22-Aug | boat |
| Cooking | 3-Jun | boat | 25-Jul | boat | 14-Aug | boat |
| Driedmeat | 4-Jun | kayak | 3-Jul | kayak | 31-Jul | boat |
| Ethel | 11-Jun | boat | 29-Jul | boat | 21-Aug | boat |
| Fork | 5-Jun | kayak | 27-Jul | boat | 19-Aug | boat |
| Frog | 15-Jul | boat | ----- | ----- | ----- | ----- |
| Garner | 30-May | scope | 27-Jun | scope | 15-Aug | boat |
| Gull | 26-Jun^1^ | boat | ----- | ----- | ----- | ----- |
| Hastings | 4-Jun | boat | 21-Jun | kayak^2^ | 16-Jul | canoe |
| Ironwood | 12-Jun | boat | 22-Jul | kayak | 18-Aug | boat |
| Isle | 24-Jun | boat | 23-Jul | boat | 11-Aug | boat |
| Kinosiu | 18-Jun | kayak | 21-Jul | kayak | 19-Aug | boat |
| Lac La Biche | 21-Jul | kayak | 6-Aug | boat | 19-Aug | boat |
| Lac la Nonne | 29-May | scope | 13-Jul | kayak/scope | 4-Aug | boat |
| Lac Sante | 9-Jun | kayak | 10-Jul | scope | 15-Aug | boat |
| Lac Ste. Anne | 26-May | kayak | 23-Jul | boat | 8-Aug | boat |
| Lesser Slave | 19-Jun | kayak^2^ | July^3^ | boat | ----- | ----- |
| Manatokan | 6-Jun | kayak | 27-Jul | boat | 18-Aug | boat |
| Missawawi | 26-Jun | kayak | 30-Jul | boat | ----- | ----- |
| Moose | 19-Jun | boat | 4-Jul | helicopter | 18-Aug | boat |
| Muriel | 8-Jul | boat | 6-Aug | boat | 17-Aug | boat |
| Murray | 12-Jun | canoe | ----- | ----- | ----- | ----- |
| North Buck | 17-Jul | boat | 5-Aug | boat | 25-Aug | boat |
| Pigeon | 13-Jun | boat | ----- | ----- | ----- | ----- |
| Pine | 3-Jul | kayak | 1-Aug | boat | 20-Aug | boat |
| Reita | 10-Jun | boat | ----- | ----- | ----- | ----- |
| Sandy | 26-May | kayak | 25-Jun | kayak | 4-Aug | boat |
| Seven Persons | 12-Jun | scope | ----- | ----- | ----- | ----- |
| Thunder | 28-May | kayak | 19-Jun | boat | 12-Jul | kayak |
| Utikuma | 12-Aug | boat | 19-Aug | boat | 27-Aug | boat |
| Wabamun | 1-Jun^1^ | boat | 23-Jun | boat | 25-Jul | boat |
| Winefred | 2-Jul | helicopter | ----- | ----- | ----- | ----- |
| Wolf | 28-Jun | kayak | 26-Jul | boat | 22-Aug | boat |

Appendix S1: Dates and survey methods (Motored boat, kayak or canoe, spotting scope) for 2008 western grebe presence/absence surveys in Alberta. ^1^Survey completed within week of specified date ^2^Colony check/nest count ^3^Survey conducted mid-July by Alberta Environment and Sustainable Resource Development
